# Subsequent Injury Risk After Return-to-Play From Lower-Extremity Muscle Injuries in Professional Male Football (Soccer)

**DOI:** 10.1177/23259671261449235

**Published:** 2026-07-09

**Authors:** Guangze Zhang, Michel S. Brink, Dominik Szymski, Lorenz Huber, Werner Krutsch, Volker Alt, Karen aus der Fünten, Tobias Tröß, Tim Meyer, Koen A.P.M. Lemmink, Anne Hecksteden

**Affiliations:** † Institute of Sports and Preventive Medicine, Saarland University, Saarbrücken, Germany; ‡ Department of Human Movement Sciences, University of Groningen, University Medical Center Groningen, Groningen, The Netherlands; § Department of Trauma Surgery, University Medical Centre Regensburg, Regensburg, Germany; ‖ FIFA Medical Centre of Excellence, University Medical Centre Regensburg, Regensburg, Germany; ¶ SportDocs Franken, Nürnberg, Germany; # University Sports, Stuttgart University, Stuttgart, Germany; ** Institute of Sport Science, University of Innsbruck, Innsbruck, Austria; †† Institute of Physiology, Medical University of Innsbruck, Innsbruck, Austria; Investigation performed at Saarland University, Saarbrücken, and University Medical Centre Regensburg, Regensburg, Germany

**Keywords:** football, muscle injury, return to play, subsequent injury, survival analysis

## Abstract

**Background:**

Injuries to the upper extremity account for nearly half of the overall injury burden in professional football (soccer), with incidence rates differing across major muscle groups under both acute and overuse conditions. Importantly, they are commonly associated with an increased susceptibility to subsequent injury, especially in the weeks after return to play (RTP). However, the specific post-RTP risk trajectories after different lower-extremity muscle injuries remain insufficiently characterized.

**Purpose:**

To investigate the risk trajectory for noncontact subsequent injury after returning from major lower-extremity muscle injuries in professional football players, differentiating acute from overuse index injuries.

**Study Design:**

Descriptive epidemiology study.

**Methods:**

Prospectively collected injury data from clubs in the first and second German Bundesliga over 3 seasons (2022-2023 to 2024-2025) were used for time-to-event analysis. Index injuries (ie, injuries from which players returned to play during the study period) of interest included hamstring, quadriceps, adductor, and calf injuries. Acute and overuse index muscle injuries were analyzed separately across these locations. Noncontact time-loss subsequent injuries occurring in the same season as RTP were considered the event of interest. Built upon previously published methodology, the Kaplan-Meier survival function was used to estimate the cumulative hazard function, from which the continuous hazard function was derived.

**Results:**

During the 3 analyzed seasons, 374 acute and 304 overuse index injuries were identified at the 4 lower-extremity muscle groups. Observed noncontact subsequent-injury rates were 27.9%, 26.2%, 39.3%, and 21.2% after acute hamstring, quadriceps, adductor, and calf index injuries, respectively, and 28.1%, 27.7%, 27.2%, and 27.4% for overuse index injuries. While the time course of noncontact subsequent injury risk globally aligned with previous findings, differences between muscle groups were observed. Players returning from acute hamstring and adductor injuries showed elevated risk, which diminished within approximately 12 and 4 weeks, respectively, and leveled off thereafter. By contrast, a delayed risk peak around 3 weeks after RTP was found for overuse calf index injuries.

**Conclusion:**

This descriptive study further confirms the time-varying nature of subsequent injury risk for specific lower-extremity muscle injuries. The observed differences in risk trajectories across muscle groups highlighted the need for careful RTP decision-making based on each index injury.

Determined by the nature of the sport, football (soccer) players face a high injury risk during training and matches, especially to the lower extremities.^2,11,41^ Muscle-related injuries account for approximately half of injury occurrences in professional football,^2^ with thigh (hamstring, adductors, and quadriceps) and calf as the most affected^7,19^ muscle groups (>90%). After return to play (RTP) from lower-extremity injuries, an elevated risk of subsequent injury has been reported, including both recurrent (same type and site) and new (different) injuries.^28,41^ While incomplete tissue healing and regeneration may predominantly contribute to reinjury risk,^24,25,34^ subsequent injury risk may increase due to deconditioning, persistent functional deficits, and psychological readiness.^1,9,30^ Notably, reinjuries account for only 7% to 18% of all subsequent injuries,^10,37,40^ with most recurrences occurring within the first weeks after RTP.^17,31,35,42^ In contrast, overall subsequent injury risk may remain elevated for a longer post-RTP period.^32,39^ To minimize the impact of successive injuries on individual athleticism and team success, recent studies have emphasized the importance of investigating subsequent injury risk.

Despite existing knowledge of a higher injury risk after RTP, previous studies on sport injury risk^5,17-19^ often reported average incidence over certain follow-up periods, potentially obscuring the temporal distribution of subsequent injury. Consequently, the averaged risk is highly dependent on the chosen follow-up duration and, in some instances,^23^ may be overturned by a different follow-up period. Moreover, the number of players who have not sustained a subsequent injury also decreases over time, as each injury reduces the cohort. A shrinking cohort could lead to a decrease in injury incidence despite a constant or even slightly increasing underlying injury risk.

In contrast, time-to-event analysis accounts for the decreasing number of individuals who remain free of subsequent injuries. In particular, analysis using the Kaplan-Meier model^23,29,44^ enables the identification of potentially time-varying, period-specific subsequent injury risk (hazard) and the estimation of a continuous risk trajectory, whereas approaches such as the Cox proportional hazard model^4,23^ assume a constant hazard ratio between subgroups. A continuous trajectory of subsequent injury risk after returning from general football injuries was previously presented to inform post-RTP player management.^44^ However, further knowledge of this at the level of index injury diagnosis remains to be investigated. This should include heterogeneous body tissues (eg, muscles) with distinct natures and functions.

Lower-extremity muscle injuries are heterogeneous, with hamstring injuries accounting^7^ for 37% of all muscle injuries, followed by adductor (23%), quadriceps (19%), and calf injuries (13%). Acute lower-extremity muscle injuries are characterized by a sudden onset. In contrast, overuse injuries develop gradually and account for a larger proportion of injuries within the adductor group (42%) than within the hamstring (30%), quadriceps (26%), or calf (28%) groups.^7,8,14,43^ Despite distinct injury mechanisms, acute and overuse lower-extremity injuries were often analyzed collectively, with limited evidence on their respective post-RTP events.^7,16,35^

Given the limited sample size of reinjuries and the considerable influence of random chance in contact injuries,^20,21^ the present study aimed to characterize the continuous risk trajectory of noncontact subsequent injury after RTP from hamstring, quadriceps, adductor, and calf muscle injuries, differentiating acute from overuse index injuries.

## Methods

### Team-Reporting Injury Collection

A prospective injury collection (Bundersligaregister) was conducted over 3 consecutive seasons (2022-2023 to 2024-2025) with professional football clubs in Germany (first and second Bundesliga), in collaboration with the Deutsche Fußball Liga (DFL) and the Verwaltungs-Berufsgenossenschaften (VBG; for the 2022-2023 season). In total, 1475 players participated, including 796 players from 23 clubs in 2022-2023, 881 from 27 clubs in 2023-2024, and 748 from 23 clubs in 2024-2025. Time-loss injuries were prospectively registered by medical staff from each club in accordance with international guidelines on football injury collection,^3,12^ through the REDCap platform (REDCap, Research Electronic Data Capture, Vanderbilt University), a bespoke platform for this cohort study. According to the mode of onset, team medical staff classified injuries upon reporting as acute (sudden onset and without repetitive mechanism) or overuse (gradual onset and/or attributable to underlying repetitive microtrauma).^3,12,14^ This commonly used binary classification is not always clear-cut and may therefore result in some loss of information. The injury mechanism was specified as (1) contact injury with a direct contact at the injured location, (2) indirect contact injury caused by a contact at another location, and (3) noncontact injury.^3^ Data entry was weekly checked for plausibility by an orthopaedic surgeon (D.S.). Written and informed consent was obtained from all first-team players from participating clubs. This cohort study was approved by the ethics committee at the Universität Regensburg (ID: 22-2919-101).

### Preprocessing and Censoring

**Figure 1.**
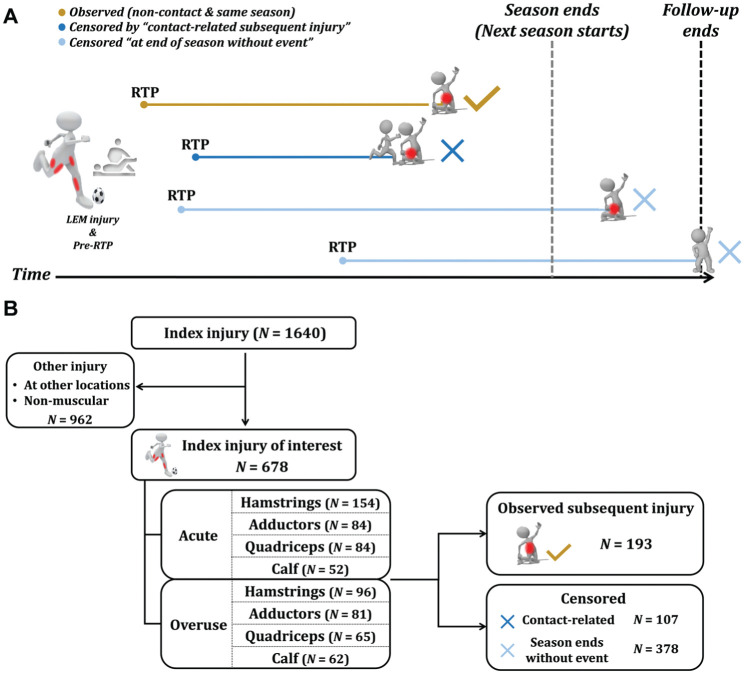
(A) Identification of observed subsequent injuries after returning from index injuries of interest (reproduced from Zhang et al^44^). (B) Flowchart of index injury inclusion as well as observed and censored subsequent injuries. LEM, lower-extremity muscles.

In the present analysis, index injuries of interest included hamstring, quadriceps, adductor, and calf injuries. Acute and overuse index muscle injuries were analyzed separately across these locations (Figure 1). Specifically, index injury diagnoses comprised contusion, painful muscle spasm, muscle strain, muscle fiber tear (with/without the involvement of fascia/musculotendinous junction), muscle bundle tear (with/without the involvement of fascia/musculotendinous junction), muscle rupture (with/without the involvement of fascia/musculotendinous junction), tendon rupture, tendinopathy, and other overuse muscle/tendon injury.^3,12^ Diagnostic methods, such as ultrasound, radiography, magnetic resonance imaging (MRI), computed tomography, and laboratory testing (eg, blood tests), were adopted by medical staff accordingly.

An index injury of interest is followed by rehabilitation, RTP, and potentially a subsequent injury (Figure 1). RTP in this study was defined as full availability for training and competition.^17,44^ Given the relatively low predictability of physical contact in football, only noncontact, time-loss injuries were considered as subsequent injuries. For each individual, the first injury during the study period was considered the index injury for the following one, the second injury the index injury for the third, and so on. The last injury in each individual's record was also reserved for time-to-event analysis (Figure 1).

In time-to-event analysis, censoring refers to an abbreviated length of follow-up due to the end of the follow-up period or to reasons unrelated to the target event.^29^ As previously published,^39,44^ 2 types of censoring were considered in this study. First, because this study emphasized the noncontact subsequent injury risk after RTP, contact-related subsequent injuries were censored (Figure 1). Second, given the different training and match exposure during the summer break compared to in-season, scenarios without a subsequent injury in the same season as RTP were censored at the end of the season (Figure 1). Altogether, a subsequent injury of interest was confirmed by being observed in both categories, that is, noncontact subsequent injury occurring in the same season as RTP. The end of each season was defined as the date of the last official match in the Bundesliga, the Bundesliga relegation playoffs, or the DFB-Pokal. When applicable, this definition was adjusted for clubs whose participation in Union of European Football Associations (UEFA) club competitions extended their season beyond the German domestic season. The knit R Markdown file for data analysis is available in an open-access repository (https://osf.io/x9asm/overview?view_only=61e859b6cc844c96aa0e9043ad2e151e).

### Hazard Function

The approach to deriving the continuous hazard function was primarily based on the methodology of an earlier study.^44^ First, time-to-event data with censoring information were used to fit the Kaplan-Meier (KM) model^29^ to estimate the survival function, the probability of remaining free of noncontact subsequent injury at each time *t_j_* after RTP. Second, the KM survival function serves for calculating the cumulative hazard function based on an established mathematical relationship.^38^ The cumulative hazard function provides a pivot to retrieve the continuous hazards as its first derivative (local slope).^38^

Third, to derive fine-grained curves, a cubic spline model (*df* = 5) was employed instead of a 10th-degree polynomial,^44^ fitting cumulative hazards as the response and time as the explanatory variable. Subsequently, predictions were made for the first 100 days after RTP. Lastly, the rate of change in predicted cumulative hazards was calculated as the instantaneous risk at each day after RTP (the continuous hazard function), that is, the risk trajectory for subsequent noncontact injury.

Statistical differences across survival functions were tested using the log-rank test.^27^ It is noteworthy that there is no necessary correspondence between statistical inference on survival functions and that on hazard functions. Observed differences across hazard curves have not been statistically tested and should be interpreted as descriptive.

## Results

### Epidemiology

In the analyzed injuries (N = 1640) over 3 seasons (Figure 1B), 678 (41.3%) affected these 4 muscle groups, including 250 (15.2%) occurring at hamstrings, 165 (10.1%) at adductors, 149 (9.1%) at quadriceps, and 114 (7%) at calf muscles. Details of specific diagnoses by muscle group are provided in Supplementary Digital Content 1. Of these, 374 were classified as acute and 304 as overuse index injuries affecting the 4 lower-extremity muscle groups (Figure 1B). Among the included 678 lower-limb muscle injuries, 635 were evaluated with ultrasound and/or MRI after clinical examination.

#### Acute Lower-Extremity Muscle Injury and Its Subsequent Injury

As the most prevalent muscle injuries, acute hamstring injuries (N = 154) led to 43 (27.9%) noncontact subsequent injuries that occurred in the same season as RTP (observed subsequent injury). Recurrences (N = 18) accounted for 41.9% of observed subsequent injuries after acute hamstring injuries. Additionally, 84 acute adductor injuries were followed by 33 (39.3%) observed subsequent injuries (3 recurrences; 9.1%), 84 acute quadriceps injuries by 22 (26.2%) observed subsequent injuries (2 recurrences; 9.1%), and 52 acute calf injuries by 11 (21.2%) observed subsequent injuries (with no recurrences).

**Figure 2.**
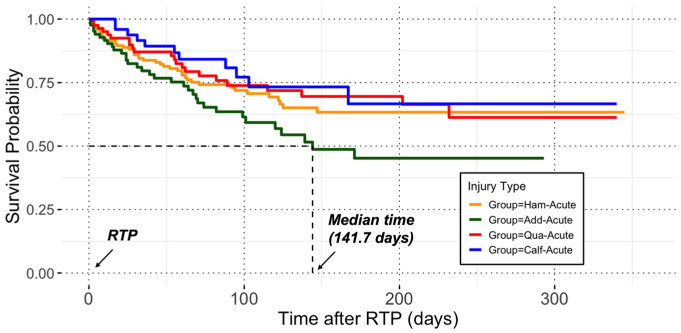
Probability of remaining free of noncontact subsequent injury after acute hamstring (ham), adductors (add), quadriceps (qua), and calf muscle injury. RTP, return to play.

Figure 2 showed a median survival time of 142 days after players’ RTP from acute adductor injuries. In contrast, >50% of individuals remained free of an observed subsequent injury after returning from acute hamstring, quadriceps, and calf injuries. There was no significant statistical difference in survival curves across acute lower-extremity index injuries (*P* = .08).

#### Overuse Lower-Extremity Muscle Injury and Its Subsequent Injury

**Figure 3.**
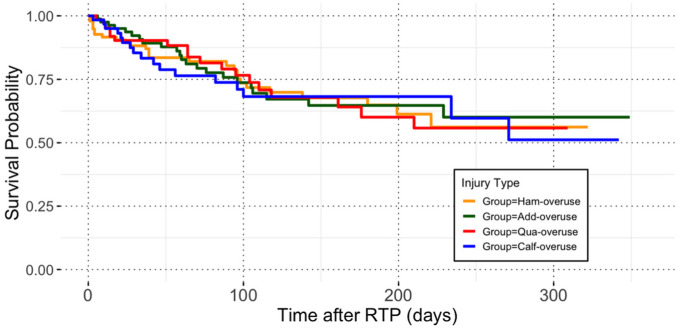
Probability of remaining free of noncontact subsequent injury after overuse hamstrings (ham), adductors (add), quadriceps (qua), and calf muscle injury. RTP, return to play.

Among 304 RTP cases followed an overuse lower-extremity muscle injury, 96 hamstrings index cases were followed by 27 (28.1%) observed subsequent injuries (8 recurrences; 29.6%), 81 adductor cases by 22 (27.2%) observed subsequent injuries (2 recurrences; 9.1%), 65 quadriceps cases followed by 18 (27.7%) observed subsequent injuries (3 recurrences; 16.7%), and 62 calf cases followed by 17 (27.4%) observed subsequent injuries (4 recurrences; 23.5%). No significant difference in survival curves was found across overuse lower-extremity index injuries (*P* = .98). The median survival probability was not reached in any of the overuse groups (Figure 3).

### Risk Trajectory After *Acute* Lower-Extremity Muscular Injury

**Figure 4.**
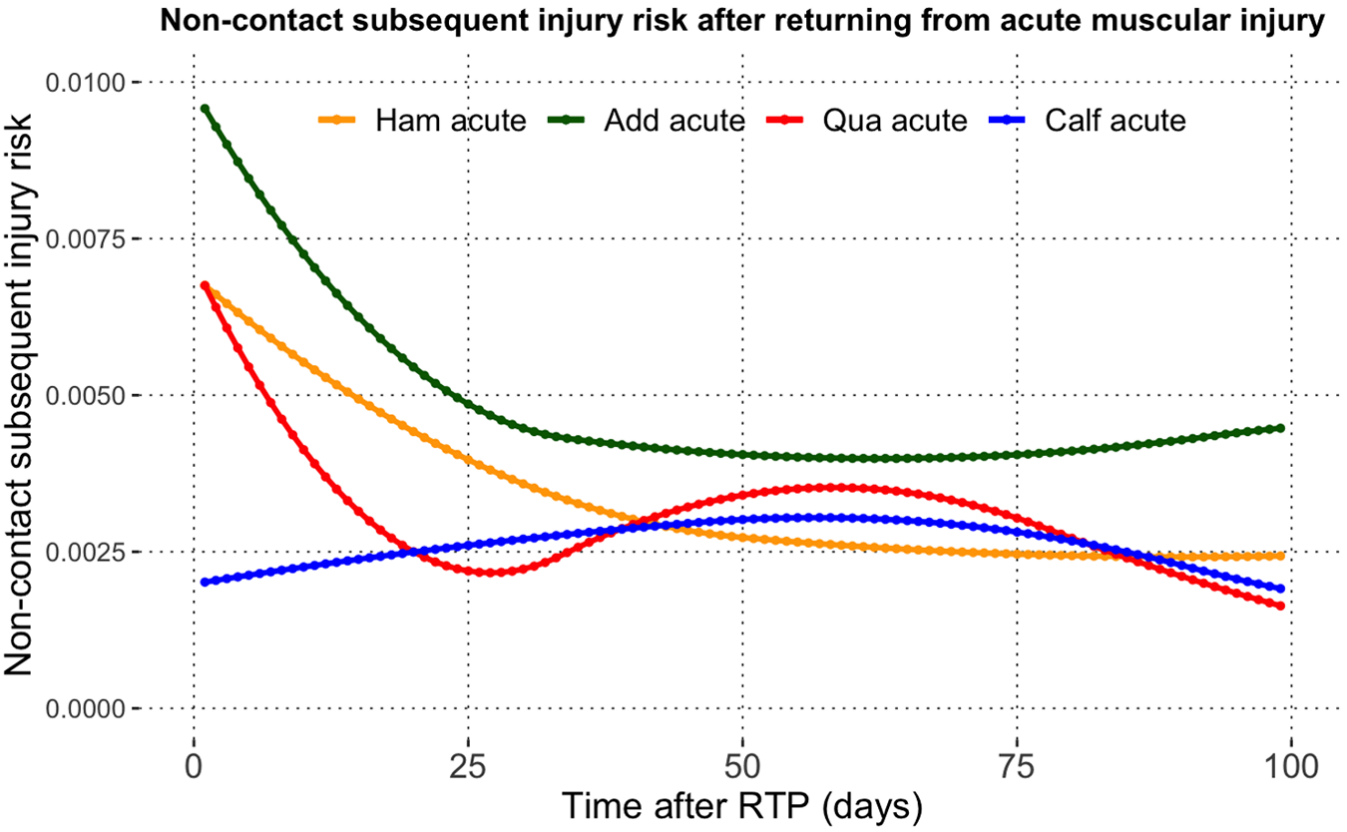
Noncontact subsequent injury risk after returning from acute hamstring (ham), adductor (add), quadriceps (qua), and calf muscle injury. RTP, return to play.

As players returning from acute hamstring injuries, the elevated noncontact subsequent injury risk steadily decreased over the following 50 days (Figure 4). A similar overall shape of the hazard curve was found for acute adductor injury. However, the risk shortly after RTP was about twice the baseline, which then plunged by half within about 4 weeks after RTP and remained higher than for other acute lower-extremity muscle injuries. A major decline was also observed within the 4 weeks after returning from acute quadriceps injuries, followed by a temporary minor risk increment. By comparison, acute calf index injuries showed a different pattern, with a low risk in the first days after RTP that remained relatively consistent throughout the post-RTP period.

### Risk Trajectory After *Overuse* Lower-Extremity Muscular Injury

**Figure 5.**
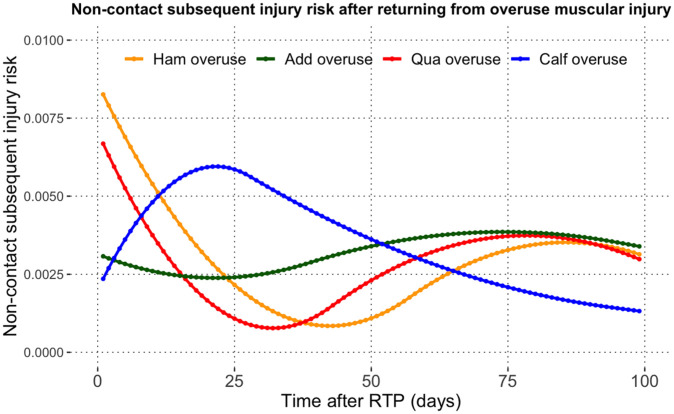
Noncontact subsequent injury risk after returning from overuse hamstring (ham), adductors (add), quadriceps (qua), and calf muscle injury. RTP, return to play.

Comparable risk trajectories of noncontact subsequent injury were observed after overuse hamstring and quadriceps injuries (Figure 5). Both showed a relatively higher risk than the other 2 muscle groups as players returned to full availability. The risk diminished to its lowest level within approximately the first month, then increased slightly and fluctuated over time.

Conversely, players returning from overuse calf injuries experienced a delayed risk peak around 3 weeks after RTP. While it declined progressively thereafter, a higher risk than that of the other 3 muscle groups persisted until around day 50 after RTP. After adductor overuse injuries, the subsequent noncontact injury risk tended to be dynamic and generally lower during the post-RTP period.

## Discussion

This descriptive study confirmed the time-varying nature of subsequent injury risk and the overall hazard-curve patterns recently identified in male professional football.^44^ By distinguishing acute from overuse index injuries, it further showed that post-RTP risk evolved differently across specific index lower-extremity muscle injuries. In contrast, previous work often pooled time-loss index injuries in the analysis. Players returning to full football activity after acute hamstring, adductor, and quadriceps injuries faced an elevated risk of noncontact subsequent injury shortly after RTP, with the acute adductor and quadriceps group showing a rapid decline in risk within the first 4 post-RTP weeks. Overuse of lower-extremity muscles was associated with greater risk variability than in acute index groups. The hazard-curve approach implemented here characterized the continuous risk trajectory over the post-RTP period, in contrast to previous studies^5,18,19^ that reported averaged incidence over certain follow-up periods. Statistical differences across hazard curves were not formally tested because no established method exists for this purpose. Lastly, consistent with the reported differences in time-to-event between subsequent injury^32,39^ and reinjury,^17,31,35,42^ the present study observed a prolonged elevation in subsequent injury risk compared with previous findings focusing specifically on reinjury, reflecting the fact that our study included all noncontact subsequent injuries.

### RTP From *Acute* Lower-Extremity Muscle Injuries

#### Time to Subsequent Injury

In the present time-to-event analysis, elevated noncontact subsequent injury risk was observed shortly after RTP from acute hamstring, adductor, and quadriceps injuries, and this risk declined within the first 50 days. Risk following acute calf muscle injuries appeared relatively low and stable throughout the post-RTP period. Similarly, in Australian football, Stares et al^39^ found that the majority of subsequent injuries occurred within the first 50 days after lower-extremity muscle injuries. However, no muscle-specific time-to-event patterns were provided.

More specifically, after acute hamstring injuries, the risk decreased by >50% within the first month and leveled off around 50 days. This supports previous findings that hamstring injury is associated with an increased risk of subsequent quadriceps^32,33^ and calf^33^ strains within the first 8 weeks after RTP.

Players returning from acute adductor injuries experienced a higher (than other acute groups) and declining noncontact subsequent injury risk during the first 4 weeks. Thereafter, the risk remained low, with a slight upward trend around 3 months after RTP. Although Serner et al^37^ focused on acute adductor reinjuries, they also reported another wave of subsequent injuries approximately 4 months after RTP after acute adductor injuries, which aligns with the later increase observed here.

Similar to the acute adductor group, the risk decreased drastically within the first four weeks after RTP after acute quadriceps injuries, compared with a more gradual decline in the acute hamstring group. Beyond reinjury risk, elevated subsequent injury risk after quadriceps injury may also reflect increased post-RTP injury risk at other locations (eg, the calf).^32^

#### Interpretations of Differences in Risk Trajectories After Acute Index Injuries

*Key Determinants.* Because the current descriptive study did not investigate the key determinants (eg, exposure during RTP^39^) that potentially contribute to the differing patterns across risk trajectories, speculative explanations remain limited. These differences may reflect, at least in part, the various functional roles and loading profiles of the 4 muscle groups in football.^6,8,11,15,26,35,36^

*Varying Proportions of Recurrences.* The varying proportions of recurrences across muscle groups may also influence the observed risk-trajectory shapes, particularly within the first post-RTP weeks. Recurrences accounted for 41.9% of observed subsequent injuries after acute hamstring injuries (vs acute adductor: 9.1% vs acute quadriceps: 9.1% vs acute calf without recurrences). It can be speculated that a large proportion of recurrences in the acute hamstring group might partly slow down the decline of noncontact subsequent injury risk. In contrast, the risk of acute adductor and quadriceps injuries diminished during the first post-RTP weeks, after reinjuries had occurred. No recurrences after acute calf injuries led to a relatively lower risk (than other acute groups) and consistent trajectory. Further research with larger sample sizes will enable characterization of risk trajectories for reinjuries, thereby scrutinizing the importance of variations in recurrence rates between muscle groups in the present work.

### RTP From *Overuse* Lower-Extremity Muscle Injuries

Overuse of lower-extremity muscle injuries was followed by greater variability in risk trajectories than acute index injuries. Post-RTP outcomes of nonacute lower-extremity muscle injuries have rarely been examined independently in previous studies.^7,13,16,35^ The methodology of the present work allowed characterization of dynamic risk trajectories after specific overuse lower-extremity muscle injuries. Differences in risk trajectories across overuse index injuries are discussed below, together with cautious interpretations.

#### Interpretations of Differences in Risk Trajectories After Overuse Index Injuries

In addition to the determinants described above, post-RTP event composition may also influence the observed risk-trajectory patterns after overuse injuries. Relatively high recurrence proportions were observed for overuse hamstring and quadriceps index injuries, which also showed elevated risk shortly after RTP, followed by a decline. By contrast, overuse adductor injuries had a lower recurrence proportion and correspondingly no pronounced early post-RTP drop in risk. The delayed risk peak observed in the overuse calf group is in line with previous reports of a delayed cluster of calf reinjuries after RTP^33^ in Australian football. However, that study did not distinguish between acute and overuse injuries.

### Strengths, Limitations, and Future Directions

With prospectively collected injury data across several seasons after medical team-reporting methodology,^9^ this study conducted time-to-event analysis at the level of injury diagnosis, distinguishing acute from overuse lower-extremity muscle injuries. Built upon the data-driven approach of a previous publication,^44^ to capture local variations in cumulative hazards while ensuring stability, the current analysis utilized a basic spline model with a low degree of freedom (*df* = 5) instead of a 10th-degree polynomial,^44^ addressing potential concerns for overfitting. By introducing an independent dataset, we observed similar risk trajectories across the 2 datasets despite differences in risk magnitude (supplemental digital content 3). Besides, each individual's last injury was reserved and censored by the season's end accordingly. This allowed us to capture all RTP scenarios and, therefore, to derive more genuine subsequent injury risk trajectories qualitatively and quantitatively.

Nevertheless, several limitations should be acknowledged. First, as an observational epidemiologic study, the analyses are descriptive and preclude causal conclusions. Second, RTP was defined as full availability for training and matches, without a standardized set of objective testing criteria across clubs. Although rehabilitation and testing are routinely used in professional football (especially in the current cohort), the specific RTP processes and decision thresholds likely varied by clubs and injuries, and may have been influenced by competitive demands, potentially leading to earlier RTP and inadequate recovery.^22,24,25,34^

Third, the current sample size does not allow analysis of a finer categorization of index injuries (eg, grade 1 vs 2 vs 3 hamstring strains, or severities by time-loss). Greater muscle damage tends to lead to longer regeneration and scar formation, which, combined with recovery time and post-RTP load management, could influence the injury risk after RTP. Beyond categorizing index injuries, future research with larger sample sizes could further differentiate recurrent (same type and site) from new (different) injuries (see supplemental digital content 2 for a breakdown of subsequent injury types) and compare their respective hazard curves. The reported proportions of recurrences among observed subsequent injuries might be influenced by end-of-season censoring. This sample-size consideration also extends to investigating risk trajectories for specific subsequent injuries (eg, ACL tears).

Fourth, although low-degree fitting was used to minimize the risk of overfitting, the limited sample sizes in each injury group (eg, acute calf) suggest that the possibility of overfitting cannot be entirely ruled out. Infrequent mid-season transfers out of the league were not explicitly modeled; however, they are expected to add only limited uncertainty to comparisons between index injury groups rather than introduce systematic bias. Moreover, while this study presented a fine-grained time course of subsequent injury risk, incorporating exposure data (training and match) is expected to provide more direct insights into the risk trajectory after RTP. This requires comprehensive collection protocols that include both detailed injury information and daily exposure data. Future research could also examine risk trajectories for those without sustaining lower-extremity injury, as a comparator to the present findings. Lastly, the generalizability of these findings might be limited to elite adult men's football. This topic warrants further research in broader populations such as women's and youth football.

## Conclusion

This descriptive study further confirmed the time-varying nature of subsequent injury risk for specific lower-extremity muscle injuries in professional male football. The observed differences in risk trajectories across muscle groups highlighted the need for careful RTP decision-making based on each index injury.

## Supplemental Material

sj-docx-1-ojs-10.1177_23259671261449235 – Supplemental material for Subsequent Injury Risk After Return-to-Play From Lower-Extremity Muscle Injuries in Professional Male Football (Soccer) Supplemental material, sj-docx-1-ojs-10.1177_23259671261449235 for Subsequent Injury Risk After Return-to-Play From Lower-Extremity Muscle Injuries in Professional Male Football (Soccer) by Guangze Zhang, Michel S. Brink, Dominik Szymski, Lorenz Huber, Werner Krutsch, Volker Alt, Karen aus der Fünten, Tobias Tröß, Tim Meyer, Koen A.P.M. Lemmink and Anne Hecksteden in Orthopaedic Journal of Sports Medicine

sj-docx-2-ojs-10.1177_23259671261449235 – Supplemental material for Subsequent Injury Risk After Return-to-Play From Lower-Extremity Muscle Injuries in Professional Male Football (Soccer) Supplemental material, sj-docx-2-ojs-10.1177_23259671261449235 for Subsequent Injury Risk After Return-to-Play From Lower-Extremity Muscle Injuries in Professional Male Football (Soccer) by Guangze Zhang, Michel S. Brink, Dominik Szymski, Lorenz Huber, Werner Krutsch, Volker Alt, Karen aus der Fünten, Tobias Tröß, Tim Meyer, Koen A.P.M. Lemmink and Anne Hecksteden in Orthopaedic Journal of Sports Medicine

sj-docx-3-ojs-10.1177_23259671261449235 – Supplemental material for Subsequent Injury Risk After Return-to-Play From Lower-Extremity Muscle Injuries in Professional Male Football (Soccer) Supplemental material, sj-docx-3-ojs-10.1177_23259671261449235 for Subsequent Injury Risk After Return-to-Play From Lower-Extremity Muscle Injuries in Professional Male Football (Soccer) by Guangze Zhang, Michel S. Brink, Dominik Szymski, Lorenz Huber, Werner Krutsch, Volker Alt, Karen aus der Fünten, Tobias Tröß, Tim Meyer, Koen A.P.M. Lemmink and Anne Hecksteden in Orthopaedic Journal of Sports Medicine
