# Supplementary material for: Subsequent Injury Risk After Return-to-Play From Lower-Extremity Muscle Injuries in Professional Male Football (Soccer)
Source: Orthop J Sports Med. 2026 Jul 9;14(7):23259671261449235. doi: 10.1177/23259671261449235 (PMC13351198; doi:10.1177/23259671261449235)
Supplement: sj-docx-1-ojs-10.1177_23259671261449235 – Supplemental material for Subsequent Injury Risk After Return-to-Play From Lower-Extremity Muscle Injuries in Professional Male Football (Soccer) [file sj-docx-1-ojs-10.1177_23259671261449235.docx]

| **Table S1-1. Diagnoses of index muscle injuries to hamstrings, quadriceps, adductors, and calf** | | | | | |
| --- | --- | --- | --- | --- | --- |
| **Index Injury Diagnosis** | | **Adductors** | **Hamstrings** | **Quadriceps** | **Calf** |
|  |  | (*N*) | (*N*) | (*N*) | (*N*) |
| **Contusion** |  | 0 | 2 | 34 | 19 |
| **Painful muscle spasm** |  | 24 | 26 | 18 | 9 |
| **Muscle strain** |  | 57 | 77 | 32 | 25 |
| **Muscle fiber tear** | (Only) | 33 | 61 | 25 | 22 |
|  | + Fascia involvement | 19 | 24 | 11 | 6 |
|  | + Musculotendinous involvement | 6 | 14 | 6 | 3 |
|  | + Fascia & musculotendinous involvement | 0 | 1 | 0 | 0 |
|  | + Tendon rupture | 0 | 0 | 0 | 2 |
| **Muscle bundle tear** | (Only) | 4 | 2 | 4 | 0 |
|  | + Fascia involvement | 0 | 3 | 3 | 2 |
|  | + Musculotendinous involvement | 1 | 9 | 0 | 0 |
|  | + Fascia & musculotendinous involvement | 0 | 1 | 0 | 2 |
| **Muscle rupture** | (Only) | 1 | 3 | 4 | 1 |
|  | + Fascia involvement | 0 | 1 | 1 | 0 |
|  | + Musculotendinous involvement | 1 | 3 | 1 | 0 |
| **Tendon rupture** |  | 4 | 4 | 2 | 8 |
| **Tendinopathy** |  | 4 | 4 | 1 | 6 |
| **Other overuse muscle/tendon injury** |  | 11 | 15 | 7 | 9 |
| ***Total*** | | 165 | 250 | 149 | 114 |
