# Supplementary material for: Subsequent Injury Risk After Return-to-Play From Lower-Extremity Muscle Injuries in Professional Male Football (Soccer)
Source: Orthop J Sports Med. 2026 Jul 9;14(7):23259671261449235. doi: 10.1177/23259671261449235 (PMC13351198; doi:10.1177/23259671261449235)
Supplement: sj-docx-2-ojs-10.1177_23259671261449235 – Supplemental material for Subsequent Injury Risk After Return-to-Play From Lower-Extremity Muscle Injuries in Professional Male Football (Soccer) [file sj-docx-2-ojs-10.1177_23259671261449235.docx]

| **Table S2-1. Categorised subsequent injuries following acute and overuse index injuries to hamstrings, quadriceps, adductors, and calf** *^a^* | | | | | | | | | |
| --- | --- | --- | --- | --- | --- | --- | --- | --- | --- |
| **Subsequent injury category** | | **Acute** | | | | **Overuse** | | | |
|  |  | **Hamstring** | **Quadricep** | **Adductor** | **Calf** | **Hamstring** | **Quadricep** | **Adductor** | **Calf** |
| **Recurrence (same site and type)** | | 18 (41.9) | 2 (9.1) | 3 (9.1) | 0 (0.0) | 8 (29.6) | 3 (16.7) | 2 (9.1) | 4 (23.5) |
| **Another lower-extremity muscle injury** | **Same side** | 9 (20.9) | 7 (31.8) | 7 (21.2) | 1 (9.1) | 3 (11.1) | 3 (16.7) | 2 (9.1) | 4 (23.5) |
|  | **Different side** | 8 (18.6) | 8 (36.4) | 11 (33.3) | 9 (81.8) | 9 (33.3) | 6 (33.3) | 9 (40.9) | 8 (47.1) |
| **Other** | | 8 (18.6) | 5 (22.7) | 12 (36.4) | 1 (9.1) | 7 (25.9) | 6 (33.3) | 9 (40.9) | 1 (5.9) |
| **Total** | | 43 | 22 | 33 | 11 | 27 | 18 | 22 | 17 |
| *^a^* Numbers in parentheses indicate the percentage of each subsequent injury type out of the total observed subsequent injuries within each index injury group | | | | | | | | | |


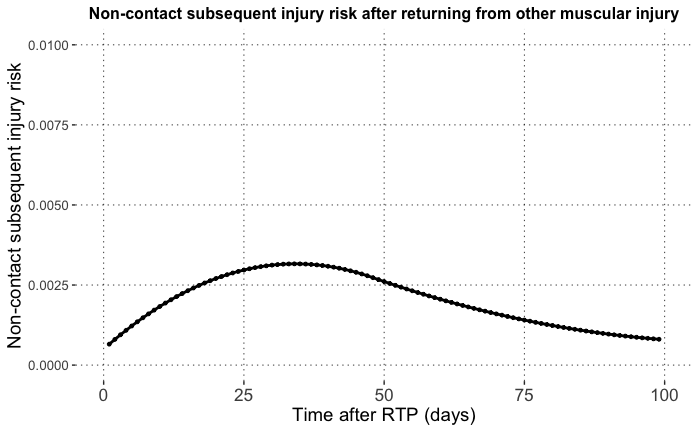


Figure S2-1. Non-contact subsequent injury risk after returning from other muscle injuries (i.e., all muscle index injuries excluding the eight muscle index injury groups presented in the main analysis, *N*=97).
