# Supplementary material for: Subsequent Injury Risk After Return-to-Play From Lower-Extremity Muscle Injuries in Professional Male Football (Soccer)
Source: Orthop J Sports Med. 2026 Jul 9;14(7):23259671261449235. doi: 10.1177/23259671261449235 (PMC13351198; doi:10.1177/23259671261449235)
Supplement: sj-docx-3-ojs-10.1177_23259671261449235 – Supplemental material for Subsequent Injury Risk After Return-to-Play From Lower-Extremity Muscle Injuries in Professional Male Football (Soccer) [file sj-docx-3-ojs-10.1177_23259671261449235.docx]

**Ancillary analysis: Verify risk magnitude and trajectory using independent datasets**

**Purpose & Materials**

Previously published media-based injury records from the *German men's 1st football league* (season 2014/15 to 2017/18)^1-3^ were used to investigate whether variations in risk magnitude between datasets would impact the overall shape of risk trajectory, thereby demonstrating the validity of hazard curves presented in the manuscript. To ensure a clear comparison between two datasets, the media-based injury dataset will be referred to as *Dataset-Media*, and the dataset presented in the manuscript will be referred to as *Dataset-Team*. It is noteworthy that Dataset-Team collection began in the season 2022/23, and therefore two datasets do not cover overlapping time periods.

Given that the Dataset-Media did not allow for analysis at the level of specific muscle sites (e.g., quadriceps etc.), the time course of non-contact subsequent injury risk after returning from acute thigh muscle injury was investigated using both datasets following the analytical pipeline presented in the main analysis. Acute thigh injuries were identified retrospectively for Dataset-Media based on injury diagnoses by a physician (AH).

**Epidemiology in two datasets**

Over seven analysed seasons, different distributions of injury severity (i.e., time loss) were found between two datasets (Figure S1-1). Minimal injuries (time loss < 4 days)^4^ accounted for 11% of all injuries in Dataset-Team, compared to 39% in Dataset-Media. During the 2014/15-2017/18 seasons (Dataset-Media), an average of 162.8$\pm$24.8 acute thigh muscle injuries occurred per season, compared to 124.3$\pm$21.5 per season during the 2022/23 to 2024/25 seasons (Dataset-Team).


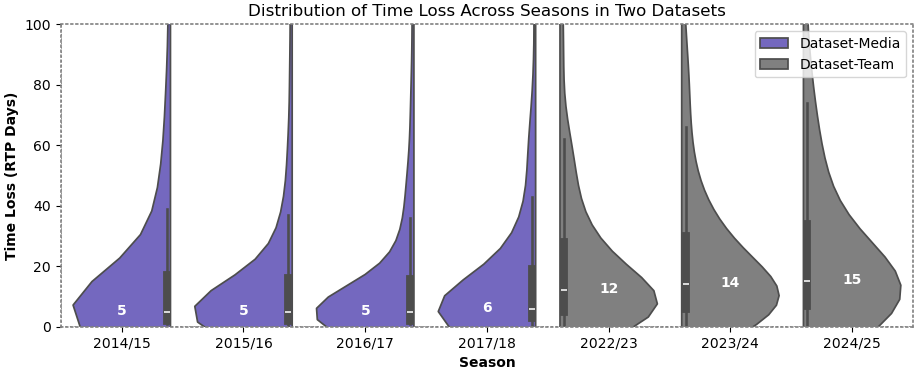


Figure S1-1. RTP time of general injuries over seasons, with the first four seasons from Dataset-Media (1^st^ Bundesliga) and last three seasons from Dataset-Team (1^st^ and 2^nd^ Bundesliga). Median time loss is annotated in white.

**Risk trajectories after return from acute thigh muscle injury**

Despite a slight difference in risk magnitude, similar risk trajectories were found in two datasets (Figure S1-2). Results from both datasets indicated that general thigh muscle injuries led to a higher non-contact injury risk shortly after RTP which gradually diminished over the post-RTP period.


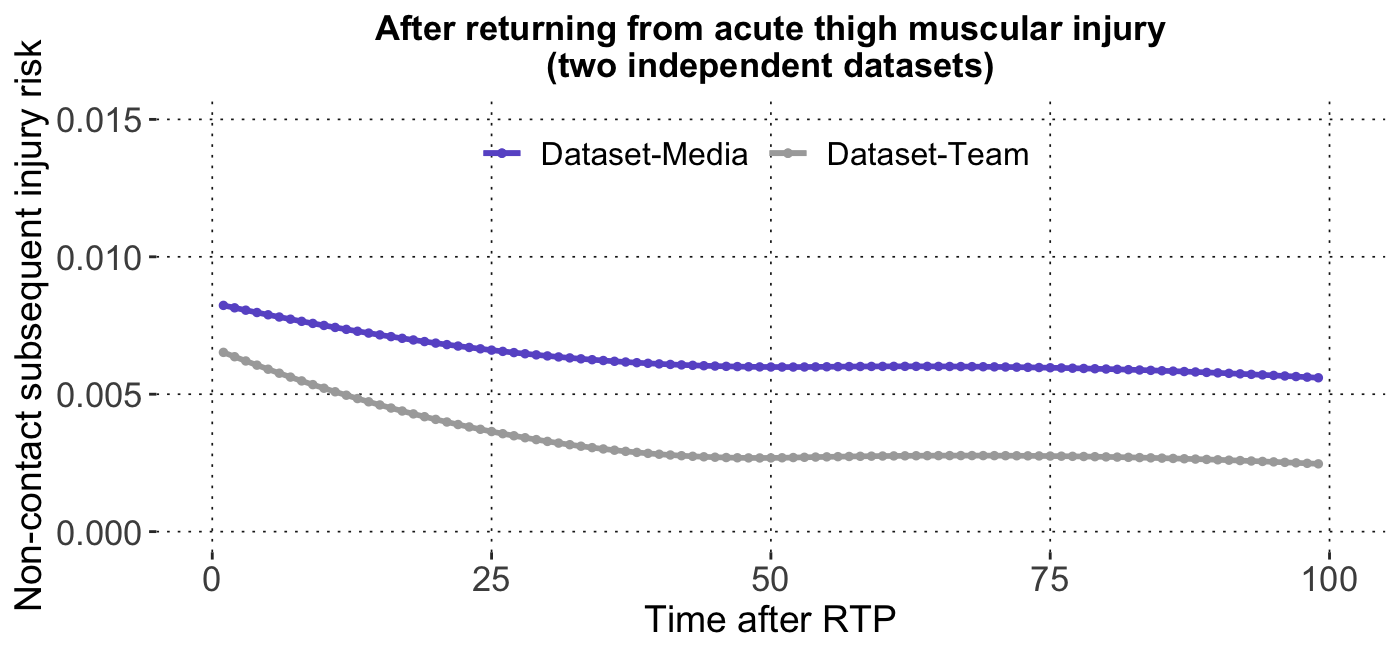


Figure S1-2. Non-contact subsequent injury risk after returning from acute thigh muscle injury from Dataset-Media (1^st^ Bundesliga) and Dataset-Team (1^st^ and 2^nd^ Bundesliga).

**Exploring differences in risk magnitude**

The slight difference in risk magnitude might result from the following reasons. First, two datasets include injuries from different populations (Dataset-Media: 1^st^ Bundesliga vs. Dataset-Team: 1^st^ and 2^nd^ Bundesliga) and seasons (Dataset-Media: season 2014/15 to 2017/18 vs. Dataset-Team: season 2022/23 to 2024/25). The injury rates in German 2^nd^ Bundesliga were previously reported lower than 1^st^ Bundesliga.^5^ Aus der Fünten, et al.^6^ reported variations in injury incidence between seasons. A lower injury incidence from the season 2019/20 onwards (4.57$\pm$0.33 per 1000 football hours) was found compared to earlier years (5.86$\pm$0.22 per 1000 football hours during season 2014/15 to 2017/18). Similarly, an independent ACL study also reported a significantly lower ACL injury rate in elite professional football since the COVID lockdown.^7^

Second, Figure S1-1 showed a smaller proportion of minimal injuries in Dataset-Team, which might contribute to the difference in risk magnitude. Therefore, the Dataset-Media was down-sampled on minimal injuries to match the severity distribution of Dataset-Team (Figure S1-3). Re-sampling reduced the difference in risk magnitude, with the overall shapes of hazard remaining unchanged (Figure S1-4).

**
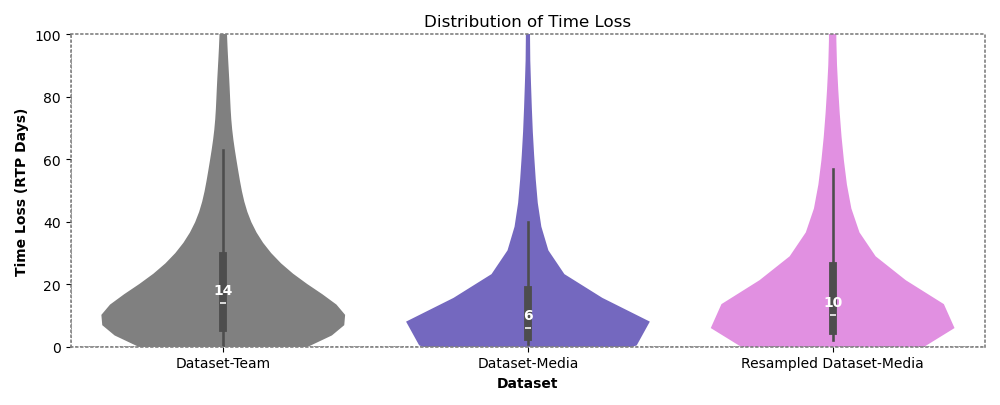
**

Figure S1-3. The distribution of time-loss in Dataset-Team, Dataset-Media, and resampled Dataset-Media.


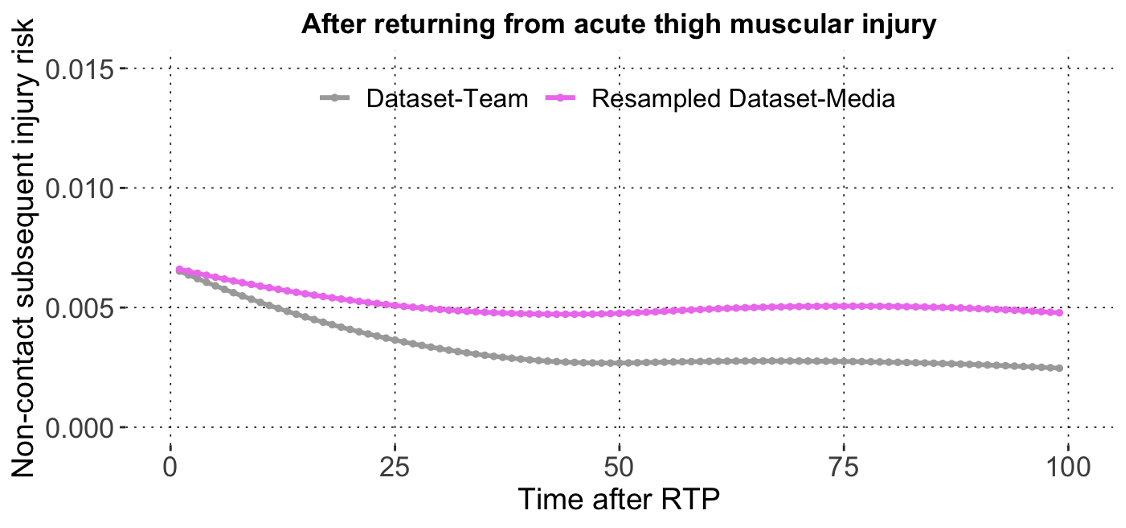


Figure S1-4. Non-contact subsequent injury risk after returning from acute thigh muscle injury, based on Dataset-Team and resampled Dataset-Media.

**Supplemental** **References**

1. Zhang G, Brink M, Tröß T, et al. The Time Course of Injury Risk After Return-to-Play in Professional Football (Soccer). *Sports Medicine.* 2024:1-9.

2. Beaudouin F, Aus der Fünten K, Tross T, Reinsberger C, Meyer T. Time Trends of Head Injuries Over Multiple Seasons in Professional Male Football (Soccer). *Sports Med Int Open.* 2019;3(1):E6-E11.

3. Krutsch W, Memmel C, Krutsch V, et al. High return to competition rate following ACL injury – A 10-year media-based epidemiological injury study in men`s professional football. *European Journal of Sport Science.* 2019;20:1-15.

4. Fuller CW, Ekstrand J, Junge A, et al. Consensus statement on injury definitions and data collection procedures in studies of football (soccer) injuries. *Br J Sports Med.* 2006;40(3):193-201.

5. Szymski D, Achenbach L, Weber J, et al. Reduced performance after return to competition in ACL injuries: an analysis on return to competition in the 'ACL registry in German Football'. *Knee Surg Sports Traumatol Arthrosc.* 2023;31(1):133-141.

6. Aus der Fünten K, Tross T, Hadji A, Beaudouin F, Steendahl IB, Meyer T. Epidemiology of Football Injuries of the German Bundesliga: A Media-Based, Prospective Analysis over 7 Consecutive Seasons. *Sports Med Open.* 2023;9(1):20.

7. Memmel C, Krutsch W, Weber J, et al. Increased rate of injuries to the anterior cruciate ligament in amateur soccer players after the COVID-19 pandemic lockdown. *Archives of Orthopaedic and Trauma Surgery.* 2024;144(9):4319-4324.
